# Effect of Plastic Film Mulching on the Grain Filling and Hormonal Changes of Maize under Different Irrigation Conditions

**DOI:** 10.1371/journal.pone.0122791

**Published:** 2015-04-13

**Authors:** Yang Liu, Juan Han, Didi Liu, Dandan Gu, Yongping Wang, Yuncheng Liao, Xiaoxia Wen

**Affiliations:** 1 College of Agronomy, Northwest A&F University, Yangling 712100, China; 2 College of Plant Protection, Northwest A&F University, Yangling 712100, China; Institute of Genetics and Developmental Biology, Chinese Academy of Sciences, CHINA

## Abstract

Plastic film mulching (PM) is widely utilized for maize production in China. However, the effect of PM on the grain yield of crops has not been established, and the biochemical mechanism underlying the increase or decrease in grain yield under PM is not yet understood. Grain filling markedly affects the grain yield. The objective of this study was to investigate the effects of PM on maize grain filling under different irrigation levels and the relationship of such effects with hormonal changes. In the present study, PM was compared with traditional nonmulching management (TN) under 220 mm, 270 mm and 320 mm irrigation amount, and the grain filling characters of the grains located in various parts of the ear and the hormonal changes in the grains were measured. The results indicated that at 220 mm irrigation, PM significantly increased the grain filling rate of the middle and basal grains and decreased the grain filling rate of the upper grains. At 270 mm irrigation, the PM significantly increased the grain filling rate of the all grains. At 320 mm irrigation, the PM only significantly increased the grain filling rate of the upper grains. The IAA, Z+ZR and ABA content in the grains was positively correlated with the grain weight and grain-filling rates; however, the ETH evolution rate of the grains was negatively correlated with the grain weight and grain-filling rates. These results show that the effect of PM on maize grain filling is related to the irrigation amount and that the grain position on the ear and the grain filling of the upper grains was more sensitive to PM and irrigation than were the other grains. In addition, the PM and irrigation regulated the balance of hormones rather than the content of individual hormones to affect the maize grain filling.

## Introduction

China is one of the largest agricultural countries in the world, which has approximately 140 million ha of agricultural lands, including a large region of dryland in the north. These agricultural lands represent approximately 56% of the nation’s total land area [1]. Maize (*Zea mays* L.) is one of the most important crops in this region, and precipitation is the major source of water for maize production in the region. However, limited and erratic precipitation, often leading to drought, is a common occurrence during the growth stage of maize and results in low yields and sometimes in total crop failure [2]. Accordingly the key to increasing the productivity of crops such as maize in this region is to maximize conservation and utilization of the soil water and achieve the largest possible increase in the water use efficiency (WUE) of the crops [3].

Many studies have indicated that plastic film mulching (PM) can conserve soil water, decrease the evaporation of soil water and substantially promote the WUE of crops [4–7]. In addition, PM can provide extra benefits, such as increasing soil temperature [8, 9]. Because of these advantages, PM is widely utilized in the dryland region of northern China.

It has been reported that, compared with traditional non-mulching management, PM could increase the grain yield of crops [5, 10, 11]. In contrast, there are reports that PM reduces, rather than increases, grain yield [12, 13]. Previous studies have suggested that the effect of PM on grain yield may be related to factors such as soil water content, soil temperature, and soil nutrition [5, 6 9 11]. However, the biochemical mechanism underlying the increase or reduction in grain yield under PM is not yet understood.

The yield potential of maize has three major components: ear number per unit area, grain number per ear and grain weight. Grain filling, the final stage of growth in cereals, in which fertilized ovaries develop into caryopses, determines the grain weight [14]. For this reason, it is important to know if and how PM affects the grain filling of maize. However, no information is available about the effects of PM on the grain filling process of maize and the underlying biochemical mechanism.

Grain filling of cereals is regulated by various factors, and plant hormones play an important role in the regulatory process. Xu et el. [15] have suggested that the zeatin and zeatin riboside (Z+ZR), indole-3-acetic acid (IAA) and abscisic acid (ABA) content in maize grains is positively and significantly correlated with the grain-filling rates and that the gibberellic acid 3 (GA_3_) content in the grains is negatively and significantly correlated with the grain-filling rates. Liu et al. [16] have suggested that IAA, ABA and ZR all increase rapidly during the early stage of high-oil corn grain development and then decline gradually until maturity. Higher endosperm cell division rates and cell numbers have been found to be associated with lower levels of GA_3_, and higher filling rates have been found to be associated with higher GA_3_. Wang et al. [17] have suggested that drought acts through the IAA, ABA and Z+ZR content to regulate grain filling in maize. Z+ZR, ABA, IAA and ethylene (ETH) have been shown to regulate grain filling in wheat and rice [13, 18–21]. In addition, brassinosteroids [22] and polyamines [23] also affect grain filling in rice. These studies have shown that hormones clearly affect grain filling in cereals, e.g., maize. However, the relationship between hormonal changes in maize grains and the grain filling induced by PM is unclear.

Previous studies have indicated that grain-filling characteristics differed substantially among grains located in different positions on the spikelets. The content of IAA and Z+ZR has been found to be higher in the superior grains than in the inferior ones at the early grain-filling stage of rice [13]. The higher ABA and lower ETH concentrations in the superior grains in wheat have been found to be associated with the higher grain filling rate in the superior grains relative to that in the inferior grains [19]. Our previous study has suggested that the inferior grains of wheat were more sensitive to water stress than superior grains [24]. However, the differences in hormonal changes among the grains located in different position on the ear and the relationship between hormonal changes in maize grains and the grain filling induced by PM is unclear. Several previous studies have suggested that the effect of PM on grain yield is related to the soil water content [5, 6, 9, 25]. In the present study, various tillage practices and various levels of irrigation were used during the maize growth stage, and the hormonal changes in the grains during grain filling were measured. The objective of this study was to investigate the relationship between the effect of PM on grain filling of maize and the soil water content and to determine how the changes in endogenous hormones in the developing grains of maize under PM are related to the grain-filling process.

## Materials and Methods

### Study site description

This study was conducted during 2012 to 2013 at the experimental station of Crop Specimen Farm in Northwest A&F University, Shaanxi Province, northwestern China, with latitude of 34°20 ' N and longitude of 108°24' E, and an elevation of 466.7m above sea level. The annual mean maximum and minimum air temperature at the site were 42°C and -19.4°C, respectively, and the annual mean temperature was 12.9°C. The total yearly sunshine duration was 2196 h and the no frost period was 220 days. The soil in the top 1.2 m was Eum-Orthrosols (Chinese soil Taxonomy), and with mean bulk density of 1.34 g cm^-3^.The Alkaline-N, NaHCO_3_-P and NH_4_OAc-K of the soil were 63.54mg kg^-1^, 19.55 mg kg^-1^ and 113.58 mg kg^-1^, respectively. The organic matter content of 0–20 cm topsoil and pH were 12.19g kg^-1^ and 7.30, respectively. In 2012, the annual mean temperature and annual mean precipitation of the experimental station were 13.6°C and 543.5 mm, respectively. In 2013, the annual mean temperature and annual mean precipitation of the experimental station were 11.7°C and 484.8 mm, respectively.

### Experimental design and treatments

The experiment was conducted in large-scale waterproof sheds. The internal shed dimensions were 32 m (length) × 15 m (width) × 3 m (height). The sheds had a transparent plastic-covered roof, and they were open on all four sides. The mobile sheds were used to control natural rainfall on a rainy day.

The experiment was a 3 × 2 (three levels of irrigation and two levels of tillage practice) factorial design with six treatment combinations. Each of the treatments had three plots as repetitions in a complete randomized block design. The length and width of each plot was 5 m×4 m, respectively. Three irrigations, consisting of applications of 220 mm, 270 mm and 320 mm, were conducted during the maize growth stage. The irrigation amounts at each stage are listed in Table 1. For each irrigation, two tillage practices, PM and traditional nonmulching management (TN), were conducted. For the PM treatment, a plastic film with a thickness of 0.04 mm was mulched on the maize field.

**Table 1:** Irrigation amount and irrigation periods.

Total irrigation amount (mm)	Irrigation stage			
	Sowing stage (mm)	Jointing stage (mm)	Large bell stage (mm)	Silking stage (mm)
220	75	55	45	45
270	75	65	65	65
320	75	75	85	85

One maize cultivar, Zhengdan 958, was grown in the field. Seeds were sown on June 14, 2012 and June 15, 2013 at a plant spacing of 60 cm× 20 cm with three seeds per hole. At the three-leaf period, one seedling was left per hole. 225 kg ha^-1^ N (in the form of urea) and 112.5 kg ha^-1^ P_2_O_5_ (in the form of calcium superphosphate) were applied. Whole P_2_O_5_ was used for the base fertilizer. Half of the N was used for the base fertilizer, and the other half was used for topdressing at the jointing stage. No precipitation fell during the maize growing season.

### Sampling and measurement

One hundred ears that silked on the same day were selected and tagged in each plot. Three tagged ears from each plot were sampled at 3-d intervals from silking to maturity. The grains of the ear were divided into three parts, on average, according to the ear length, and the grain growth was removed from the various parts of the ear. To compare grains from different growth sites on the ear, the grains from each ear were divided into upper grains, middle grains and basal grains. Half of the sampled grains were used for measurements of hormones. The other half of the grains were dried at 70°C to constant weight and weighed.

#### 1. Grain-filling process

The grain-filling data were fitted using the Richards [26] growth equation as described by Zhu et al.[27]:

$$W=\frac{A}{{\left(1+B{\text{e}}^{-kt}\right)}^{\frac{1}{N}}}$$ 1

The grain-filling rate (*G*) was calculated as the derivative of Eqn (1):

$$G=\frac{AkBe^{-kt}}{N{\left(1+Be^{-kt}\right)}^{\left(\frac{N+1}{N}\right)}}$$ 2

[*W*, the grain weight (mg); *A*, the final grain weight (mg); *t*, time after anthesis (d); *B*, *k* and *N*, coefficients determined by regression.]

The active grain-filling period was defined as the period when *W* was between 5% (*t*_1_) and 95% (*t*_2_) of A. The average grain-filling rate during this period was, therefore, calculated from *t*_1_ to *t*_2_.

#### 2. Hormones

The methods for extraction and purification of Z+ZR, GAs (GA_1_+GA_4_), IAA, and ABA were essentially identical to those described by Yang et al. [20]. A sample of approximately 0.5 g sample was ground in a mortar (on ice) with 5 mL 80% (v/v) methanol extraction medium containing 1 mmol L^-1^ butylated hydroxytoluene (BHT) as an antioxidant. The methanolic extracts were incubated at 4°C for 4 hours and centrifuged at 10,000 *×g* for 15 minutes at the same temperature. The supernatants were passed through Chromosep C18 columns and prewashed with 10 mL 100% and 5 mL 80% methanol, respectively. The hormone fractions were dried with N_2_ and dissolved in 1 mL Phosphate Buffer Saline (PBS) containing 0.1% (v/v) Tween 20 and 0.1% (w/v) gelatin (pH 7.5) for analysis with an enzyme-linked immunosorbent assay (ELISA).

The mouse monoclonal antigen and antibody against Z+ZR, GAs (GA_1_+GA_4_), IAA, ABA, and immunoglobulin G-horseradish peroxidase (IgG-HRP) used in the ELISA were produced at the Phytohormones Research Institute, China Agricultural University. The method used for quantification of Z+ZR, GAs (GA_1_+GA_4_), IAA, and ABA by ELISA has been described previously [20]. The recovery rates of IAA, Z+ZR, ABA and GAs were 87.2±3.3%, 91.6±3.8%, 90.4±3.4% and78.9±4.6%, respectively.

Ethylene evolved from the grains was determined according to Beltrano et al. [28] with modifications. Briefly, the sampled grains were placed between two sheets of moist paper for 1 h at 27°C in darkness to allow wound-induced ethylene production to subside. Each sample contained 80–100 grains. The grains were then transferred to 25-ml glass vials containing moist filter paper and immediately sealed with airtight Suba-Seal stoppers and incubated in the dark for 8 h at 27°C. A 1-ml gas sample was withdrawn through the Suba-Seal with a gas-tight syringe, and ethylene was assayed using a gas chromatograph equipped with a Porapak Q column (0.3 cm × 200 cm, 0.18–0.30 mm) and flame ionization detector (FID). Temperatures for the injection port, column and detector were kept constant at 70, 70 and 150°C, respectively. Nitrogen gas was used as a carrier at a flow rate of 40 Kpa, and hydrogen and air were used for FID at rates of 35 and 350 ml min^−1^, respectively. The rate of ethylene evolution was calculated per unit fresh weight (FW).

#### 3. Characters of ears

Twenty plants (with the exception of those on the plot border) from each plot were harvested at maturity for the determination of ear characters, i.e., the ear rows, grain number per row, and grain weight.

#### 4. Soil moisture and soil temperature

The soil moisture and soil temperature were measured at 10-d intervals after silking. The soil moisture was determined with a TDR-300 meter at a depth of 20 cm in the soil. Six measurement points were selected in each plot. The soil temperatures were measured using mercury-in-glass thermometers with bent stems. The thermometer bulbs were sunk into the ground between rows to depths of 5, 10, 15 and 20 cm. Soil temperatures were recorded daily at 8:00, 14:00 and 20:00, and the average of these three readings was calculated as the mean soil temperature.

#### 5. Leaf water potential

The leaf water potential of the flag leaves was measured at 11:00–12:00 at 10 and 20 d after silking. Well-illuminated ear leaves were chosen randomly for such measurements. A pressure chamber was used on five leaves for each treatment.

### Statistical analysis

SPSS 16.0 was used to perform an ANOVA. Data from each sampling event were analyzed separately. Means were tested with a least significant difference test at *P* = 0.05 (LSD _0.05_, $LSD_{\alpha }=t_{\alpha }s_{{\bar{y}}_{i}-\bar{y}}{}_{{}_{j}}$,${\text{s}}_{{\bar{y}}_{i}^{}-{\bar{y}}_{j}}=\sqrt{2MS_{e}/n}$). The differences in grain-filling characteristics and hormone levels between the two study years were not significant (F<1). Therefore, the data from 2013 were used to determine the grain-filling characteristics and hormone levels

## Results

### Ear characters

Irrigation significantly affected the ear characters of the studied maize. With increased irrigation, the grain number per row, 100-grain weight, and grain weight per ear of maize showed a significant increasing trend (Table 2). However, irrigation had no significant effect on the ear rows. During 2012 and 2013, the ear rows for all treatments showed no significant differences.

**Table 2:** Effects of plastic film mulching and irrigation on ear characters.

Year	Irrigation (mm)	Treatment	Eer rows	Grains per row	100-grain weight	Grain weight per ear
			(row)	(grain)	(g)	(g)
2012	220	TN	13.3a	25.7b	24.1a	82.4b
		PM	13.8a	28.2a	21.8b	84.8a
	270	TN	13.8a	30.9b	25.7b	110.0b
		PM	13.6a	34.4a	28.2a	131.9a
	320	TN	13.7a	36.1b	28.1b	139.0b
		PM	13.4a	37.9a	29.3a	148.8a
2013	220	TN	13.1a	26.1b	24.5a	83.8b
		PM	13.7a	27.9a	22.7b	86.9a
	270	TN	13.3a	30.5b	25.9b	106.1b
		PM	14.1a	33.7a	27.7a	131.6a
	320	TN	13.4a	35.4b	27.8b	131.9b
		PM	14.3a	36.8a	28.9a	152.1a

Values within a column and for the same year followed by different letters are significantly different at *P* = 0.05. PM: plastic film mulching management; TN: traditional non mulching management

PM, as well as irrigation, significantly affected the ear characters of the studied maize. PM significantly increased the grain number per row and grain weight per ear of maize at the irrigation levels of 220, 270 and 300 mm. However, PM had different effects on the 100-grain weight at different irrigation amounts. PM significantly increased the 100-grain weight at the 270- and 320-mm irrigation levels relative to the value for TN. In contrast, PM significantly decreased the 100-grain weight at the 220-mm irrigation level.

### Grain filling

**Fig 1:**
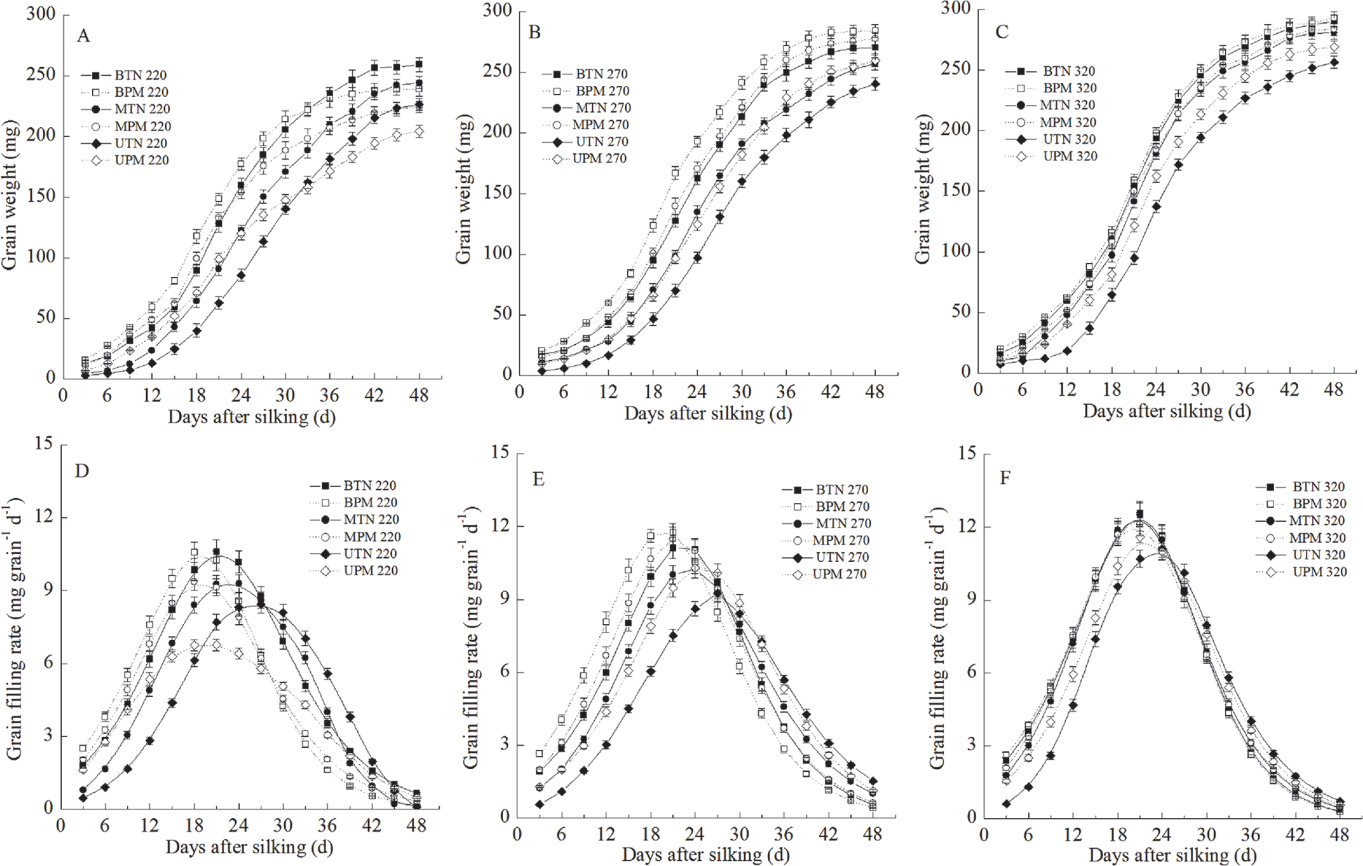
Effects of plastic film mulching on grain weights (A: 220mm irrigation; B: 270 mm irrigation; C: 320mm irrigation) and grain filling rates (C: 220mm irrigation; D: 270 mm irrigation; F: 320mm irrigation) of maize under different irrigation. B: basal grain; M: middle grain; U: upper grain. PM: plastic film mulching management; TN: traditional non mulching management. Vertical bars represent ± the standard error of the mean (n = 3).

Irrigation significantly affected grain filling. With increased irrigation, the maximum grain weights, the maximum and mean grain-filling rates and the active grain-filling period for the upper grains, middle grains and basal grains all showed an increasing trend (Fig 1, Table 3).

**Table 3:** Effects of plastic film mulching and irrigation on grain-filling characteristics of maize.

Rainfall	Grain position	Treatment	Wmax	Gmax	Gmen	D
			mg	mg per grain d^-1^	mg per grain d^-1^	d
220	Upper	PM	211.8b	6.77b	5.18b	37.1b
		TN	234.7a	8.45a	5.61a	41.3a
	Middle	PM	223.1b	9.38a	6.43a	34.7b
		TN	251.9a	9.29a	6.05b	39.2a
	Basal	PM	239.9b	10.59a	7.08a	31.6b
		TN	263.4a	10.62a	6.41b	38.1a
270	Upper	PM	262.1a	10.29a	6.23a	40.3a
		TN	246.7b	9.25b	5.72b	42.1a
	Middle	PM	282.8a	11.49a	6.88a	38.1a
		TN	260.9b	10.27b	6.14b	39.4a
	Basal	PM	287.9a	11.77a	7.22a	38.4a
		TN	274.4b	11.13b	6.47b	39.1a
320	Upper	PM	271.1a	11.59a	6.50a	41.4a
		TN	257.3b	11.01b	6.05b	42.5a
	Middle	PM	289.1a	12.28a	7.16a	39.3a
		TN	281.5a	12.52a	7.18a	39.7a
	Basal	PM	290.9a	12.50a	7.29a	38.6a
		TN	288.5a	12.59a	7.21a	39.3a

Values within a column and for the same grain type followed by different letters are significantly different at *P* = 0.05. PM: plastic film mulching management; TN: traditional non mulching management. Wmax: the maximum grain weight; Gmax: maximum grain-filling rates; Gmean: mean grain-filling rates; D: activity grain-filling period.

PM also significantly affected grain filling. At the 220-mm irrigation level, the active grain-filling periods for the upper grains, middle grains and basal grains in the PM treatment were all significantly less than that in the TN treatment. As a result, the maximum grain weights of the upper grains, middle grains and basal grains in the PM treatment were all significantly less than that of the TN treatment although PM significantly increased the mean grain-filling rates of the middle grains and basal grains. At the 270-mm irrigation level, PM significantly increased the maximum grain weights and the maximum and mean grain-filling rates of the upper grains, middle grains and basal grains relative to those in the TN treatment. At the 320-mm irrigation level, PM significantly increased the maximum grain weights and the maximum and mean grain-filling rates of the upper grains relative to those in the TN treatment; however, PM had no significant effects on these variables for the middle and basal grains.

### Hormonal changes

#### 1. IAA

**Fig 2:**
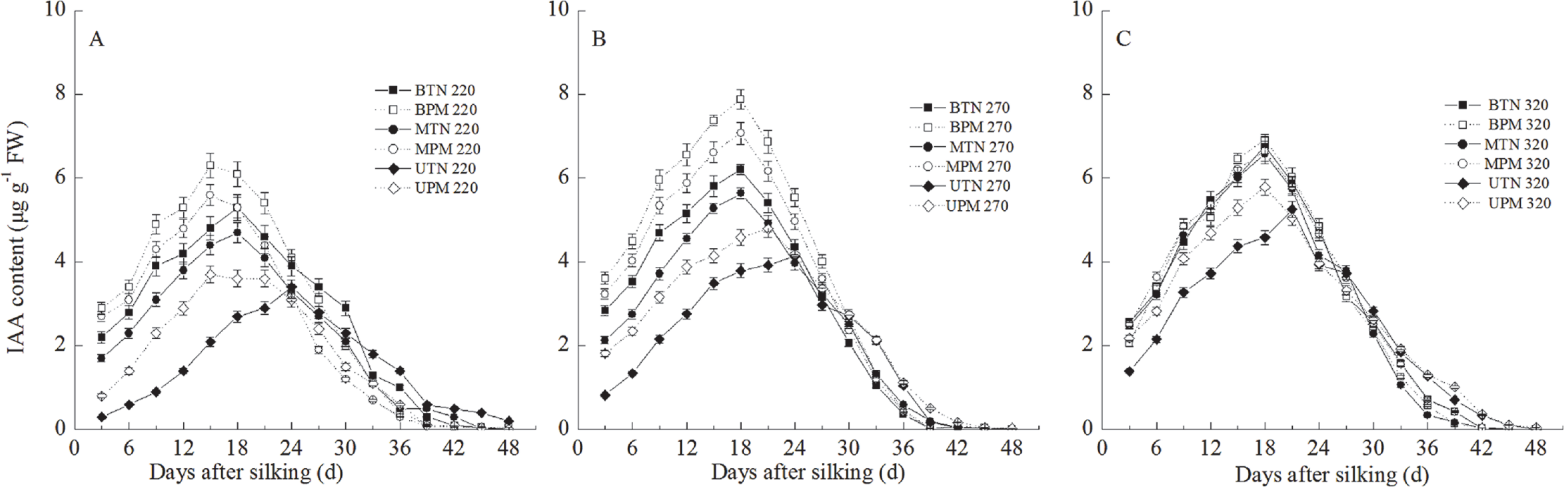
Effect of plastic film mulching on the IAA content in maize grains under different irrigation (A: 220mm irrigation; B: 270 mm irrigation; C: 320mm irrigation). B: basal grain; M: middle grain; U: upper grain. PM: plastic film mulching management; TN: traditional non mulching management. Vertical bars represent ± the standard error of the mean (n = 3).

The IAA content in the grains transiently increased at the early grain-filling stage and then decreased, and the IAA content in the basal grains and middle grains was significantly higher than that in the upper grains (Fig 2). PM had different effects on the IAA content of different grains under different irrigation levels. At the 220-mm irrigation level, PM significantly increased the IAA content in the upper grains, middle grains and basal grains. The IAA content in the upper grain, middle grain and basal grain in the PM treatment was significantly higher than that in TN at 3–24 days after silking. In addition, the peak of the IAA content in the grains in the PM treatment was advanced relative to that in the TN treatment. The IAA content in the grains of the PM treatment reached a maximum at 15 days after silking for the upper grains, middle grains and basal grains, and the IAA content in the grains of the TN treatment reached a maximum at 18 days after silking for the middle grains and basal grains and at 24 days after silking for the upper grains. At the 260-mm irrigation level, PM also significantly increased the IAA content in the upper grains, middle grains and basal grains during the early grain-filling stage. However, the peak of the IAA content in the basal grains and middle grains in the PM treatment was synchronous with that in the TN treatment. At the 320-mm irrigation level, PM also significantly increased the IAA content in the upper grains. However, PM had no significant effect on the IAA content in the basal or middle grains.

#### 2. Z+ZR

**Fig 3:**
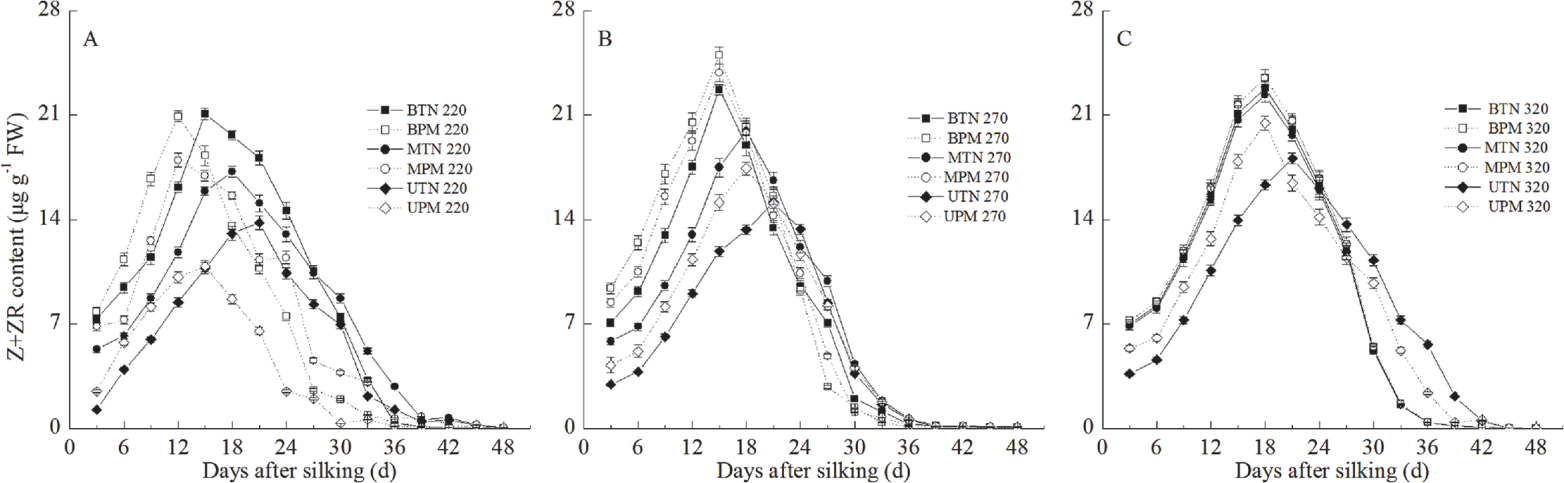
Effect of plastic film mulching on the Z+ZR content in maize grains under different irrigation (A: 220mm irrigation; B: 270 mm irrigation; C: 320mm irrigation). B: basal grain; M: middle grain; U: upper grain. PM: plastic film mulching management; TN: traditional non mulching management. Vertical bars represent ± the standard error of the mean (n = 3).

Similar to the IAA content, the Z+ZR content in the grains transiently increased at the early grain-filling stage and then decreased, and the Z+ZR content in the basal grains and middle grains was significantly higher than that in the upper grains (Fig 3). PM had different effects on the Z+ZR content in different grain categories at different irrigation levels. At the 220-mm irrigation level, PM significantly decreased the Z+ZR content in the upper grains and advanced the time of the Z+ZR peak for the upper grains. However, PM had no significant effects on the Z+ZR content in the basal grains or middle grains. PM only advanced the time at which the Z+ZR content in the basal grains and middle grains reached a maximum value. At the 270-mm irrigation level, the PM significantly increased the Z+ZR content in the upper grains, middle grains and basal grains during the early grain-filling stage. At the 320-mm irrigation level, PM also significantly increased the Z+ZR content in the upper grains. However, PM had no significant effect on the Z+ZR content in the basal and middle grains.

#### 3. ABA

**Fig 4:**
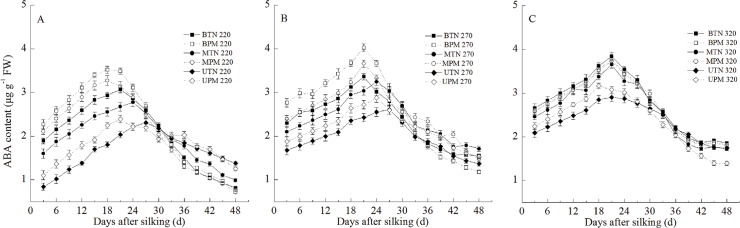
Effect of plastic film mulching on the ABA content in maize grains under different irrigation (A: 220mm irrigation; B: 270 mm irrigation; C: 320mm irrigation). B: basal grain; M: middle grain; U: upper grain. PM: plastic film mulching management; TN: traditional non mulching management. Vertical bars represent ± the standard error of the mean (n = 3).

The ABA content in the grains also transiently increased at the early grain-filling stage and then decreased, and the ABA content in the basal grains and middle grains was significantly higher than that of the upper grains (Fig 4). At the 220- and 270-mm irrigation levels, PM significantly increased the ABA content of the basal grains, middle grains and upper grains. However, PM had no significant effects on the ABA content in the basal grain and middle grains at the 320-mm irrigation level PM only significantly increased the ABA content in the upper grains at the 320-mm irrigation level.

#### 4. ETH and Gas

**Fig 5:**
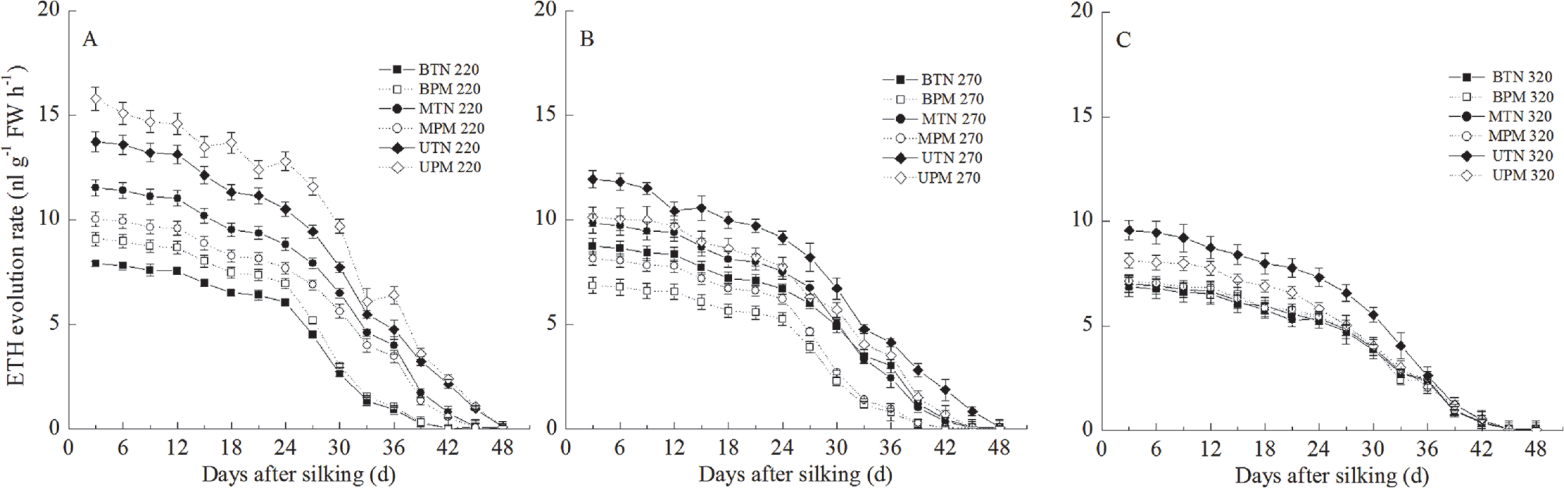
Effect of plastic film mulching on the ETH evolution rate in maize grains under different irrigation (A: 220mm irrigation; B: 270 mm irrigation; C: 320mm irrigation). B: basal grain; M: middle grain; U: upper grain. PM: plastic film mulching management; TN: traditional non mulching management. Vertical bars represent ± the standard error of the mean (n = 3).

**Fig 6:**
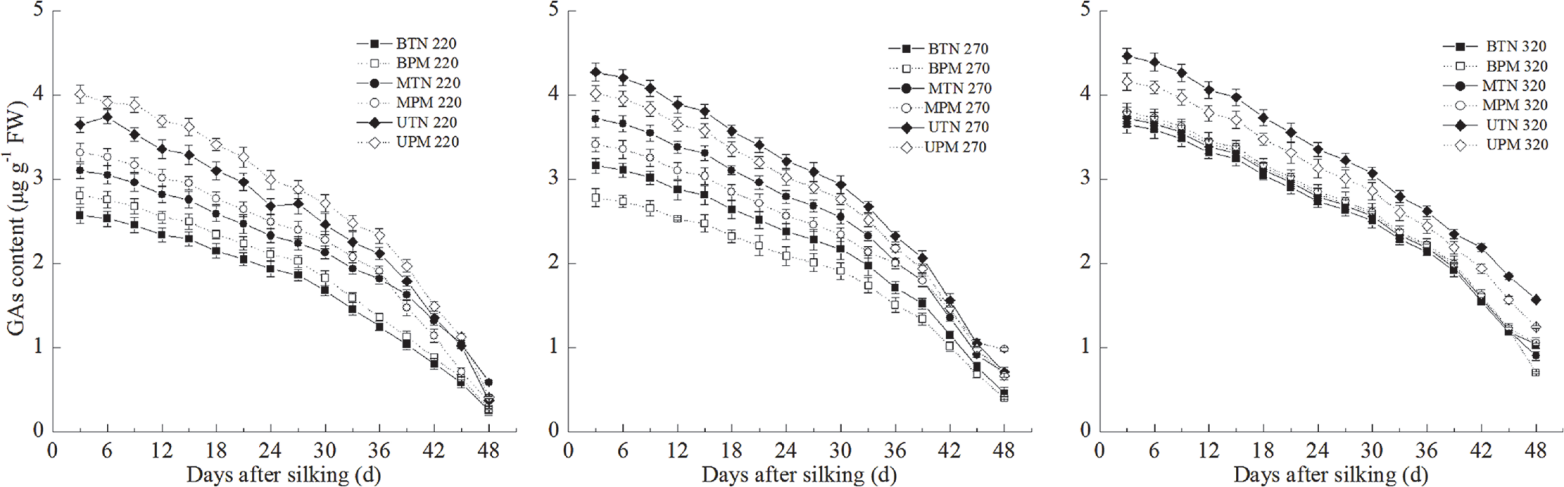
Effect of plastic film mulching on the GA_1+4_ content in maize grains under different irrigation (A: 220mm irrigation; B: 270 mm irrigation; C: 320mm irrigation). B: basal grain; M: middle grain; U: upper grain. PM: plastic film mulching management; TN: traditional non mulching management. Vertical bars represent ± the standard error of the mean (n = 3).

The ETH evolution rate and the content of GAs in the grains showed similar change patterns during grain filling. During the grain filling stage, the content of GAs and the ETH evolution rate in the grains decreased gradually (Figs 5 and 6). The ETH evolution rate and the content of GAs in the upper grains were significantly higher than those in the basal grains and middle grains during the grain-filling stage. At the 220-mm irrigation level, PM significantly increased the ETH evolution rate and the content of GAs in the upper grains, middle grains and basal grains. In contrast, at the 270-mm irrigation level, PM significantly decreased the ETH evolution rate and content of GAs in the upper grains, middle grains and basal grains. At the 320-mm irrigation level, no significant difference was observed between PM and TN for the ETH evolution rate or content of GAs in the basal grains and middle grains. However, the ETH evolution rate and content of GAs in the upper grains in the PM treatment were significantly lower than those in the TN treatment.

### Soil moisture and soil temperature

**Fig 7:**
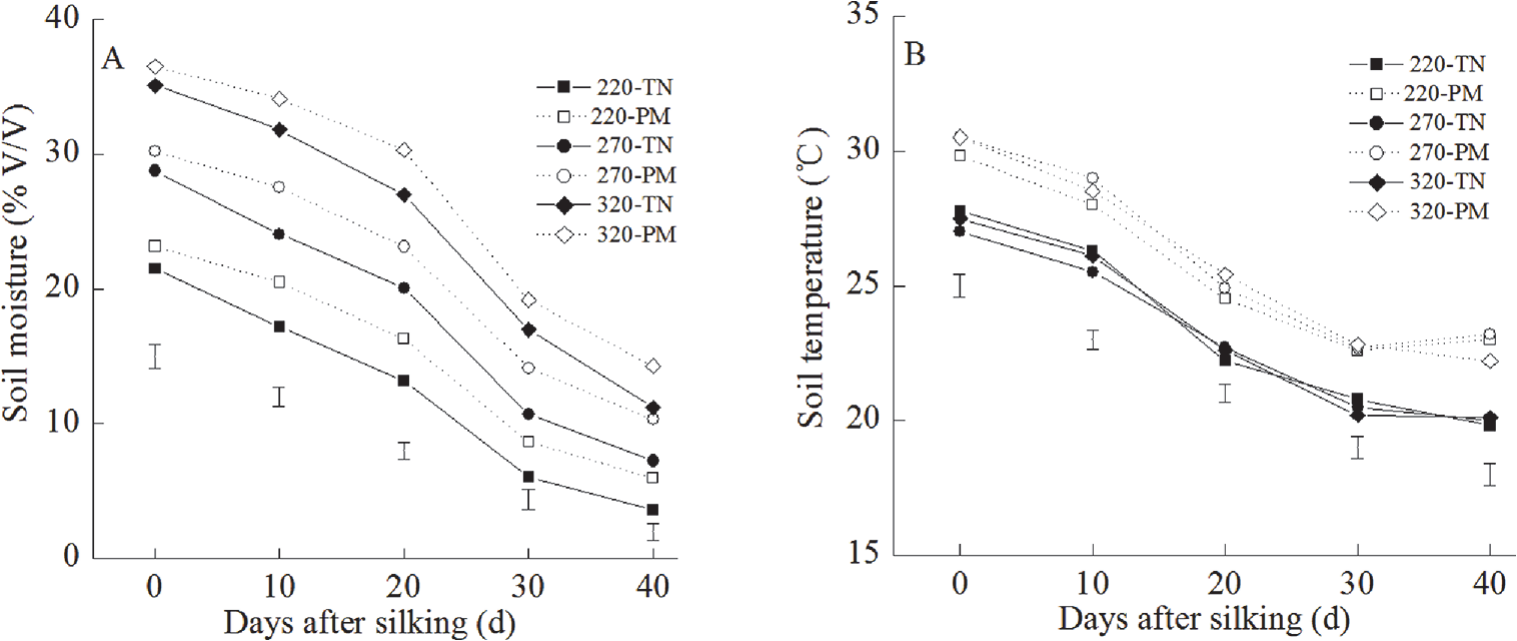
Effects of plastic film mulching on soil moisture and soil temperature during the grain-filling stage at different irrigation levels. PM: plastic film mulching management; TN: traditional non mulching management. The data of soil temperature are presented as the averages for the measured values on 5, 10, 15 and 20 cm soil layer. The vertical bars represent the LSD _0.05_.

During the grain-filling stage, the soil moisture and soil temperature showed similar patterns of change (Fig 7). During the grain-filling stage, the soil moisture and soil temperature decreased gradually. Irrigation significantly increased the soil moisture during the grain-filling stage. In contrast, irrigation had no significant effect on the soil temperature during the grain-filling stage. In addition, PM significantly increased the soil moisture and soil temperature during the grain-filling stage.

### Leaf water potential

**Fig 8:**
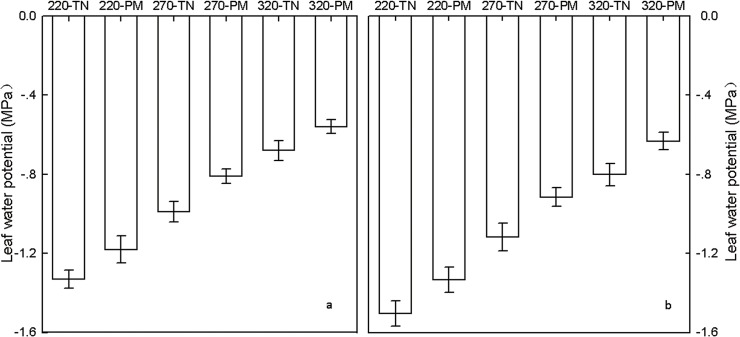
Effects of plastic film mulching on leaf water potential of ear leaf at different irrigation levels. a: 10 days after silking; b: 20days after silking. PM: plastic film mulching management; TN: traditional non mulching management. Vertical bars represent ± the standard error of the mean (n = 3).

Irrigation significantly affected the leaf water potential. With increased irrigation, the leaf water potential of the ear leaf showed an increasing trend (Fig 8). PM also significantly increased the leaf water potential. The leaf water potential in the PM treatment was significantly higher than that in the TN treatment at the 220-mm, 270-mm and 320-mm irrigation levels.

### Regression analysis of grain-filling characters and hormone levels in grains

The regression analysis showed significant differences in the grain-filling characters and hormone levels of the grains located in various parts of the ear (Table 4). For the upper grains and middle grains, the IAA, Z+ZR and ABA content was positively and significantly correlated with the maximum grain weight and the maximum and mean grain-filling rates. However, the ETH evolution rate of the grains was negatively and significantly correlated with the maximum grain weight and the maximum and mean grain-filling rates. For the basal grains, the ABA content was positively and significantly correlated with the maximum grain weight and the maximum and mean grain-filling rates, and the ETH evolution rate of the grains was negatively and significantly correlated with the maximum grain weight and the maximum and mean grain-filling rates.

**Table 4:** Correlation coefficients of peak hormone contents in maize grain with the maximum grain filling rate (G_max_), mean grain filling rate (G_mean_), maximum grain weight (W_max_) and activity grain-filling period (D) of maize.

		Wmax	Gmax	Gmen	D
Upper	IAA	0.8543*	0.9071*	0.8846*	0.4473
	Z+ZR	0.9787**	0.9922**	0.982**	0.665
	ABA	0.8807*	0.9258**	0.9002*	0.4834
	ETH	-0.9826**	-0.9947**	-0.9738**	-0.6765
	GA	0.4222	0.5192	0.3441	0.4315
Middle	IAA	0.824*	0.860*	0.876*	0.131
	Z+ZR	0.882*	0.919**	0.856*	0.379
	ABA	0.8744*	0.8861*	0.8169*	0.1046
	ETH	-0.8673*	-0.8169**	-0.8646**	-0.2917
	GA	0.6123	0.7582	0.5987	0.4396
Basal	IAA	0.316	0.3517	0.0548	0.2656
	Z+ZR	0.6228	0.6705	0.5132	0.5638
	ABA	0.8515*	0.8517*	0.8365*	0.1512
	ETH	-0.8588*	-0.8263*	-0.8578*	-0.6486
	GA	0.6233	0.856*	0.5092	0.4094
Mean	IAA	0.738**	0.825**	0.835**	-0.246
	Z+ZR	0.871**	0.918**	0.924**	-0.15
	ABA	0.772**	0.871**	0.955**	-0.291
	ETH	-0.863**	-0.965**	-0.921**	0.056
	GA	-0.076	-0.088	-0.36	0.645**

*Significant at the 0.05 probability level (for the mean grains, n = 18; for the upper grain, middle grain, basal grain, n = 6).

**Significant at the 0.01 probability level (for the mean grains, n = 18; for the upper grain, middle grain, basal grain, n = 6)).

IAA, indole-3-acetic acid; ABA, abscisic acid; Z, zeatin; ZR, zeatin riboside; GAs, gibberellins 1 plus 4; ETH: ethylene; W_max_: the final grain weight (mg); Gmax: maximum grain-filling rates; Gmean: mean grain-filling rates.

## Discussion

### Effects of irrigation and plastic film mulching on grain weight

Agricultural production in the arid regions of northern China is very dependent on water. Water has a profound impact on crop production in the region [29]. However, frequent drought is an important factor limiting crop yields because the water supply is deficient. Previous studies have suggested that PM could improve the moisture content and temperature of the soil and is an effective way to increase water availability for crop yields [4–7]. However, the effect of PM on grain yield has been in dispute. Several studies have indicated that PM significantly increases grain yield [5, 10, 11]. However, other research has found that PM reduces, rather than increases, grain yield [12, 13]. In the present study, PM significantly enhanced the ear yield of maize at the 270-mm and 320-mm irrigation levels; however, it decreased the ear yield of maize at the 220-mm irrigation level. This result means that the effect of PM on the ear yield of maize is related to the irrigation during the maize growth stage. This result is similar to those of the previous studies on maize and plastic-covered ridge and furrow planting, another plastic film mulching practice, under different rainfall levels [29].

The yield potential of maize can be dissected into three major components: ear number per unit area, grain number per ear and grain weight. A previous study has indicated that PM significantly affects the grain weight of maize [30]. In rice and wheat, the grain can be divided into superior grain and inferior grain according to the degree and rate of grain-filling. Yang and Zhang [31] suggested that the “super” rice cultivars frequently do not exhibit their high yield potential due to their poor grain-filling of the inferior grain. In the present study, we found that the effect of PM on the grain filling of maize grain is clearly related to the irrigation level. PM had different effects on the grain filling of the grains located at different positions on the ear. At the 220-mm irrigation level, PM significantly increased the grain-filling rates of the middle grains and basal grains. In contrast, PM significantly decreased the grain-filling rates of the upper grains. At the 270-mm irrigation level, PM significantly increased the grain-filling rates of the upper grains, middle grains and basal grains. At the 320-mm irrigation level, PM significantly increased the grain-filling rate of the upper grains. However, PM had no significant effect on the grain-filling rates of the upper grains and middle grains. These results indicated that the effect of PM on maize grain filling is related to the irrigation level and the position of the grain on the ear.

The results of the present study indicated that at the 220-mm irrigation level, PM significantly enhanced the grain-filling rates of the middle grains and basal grains. However, PM significantly decreased the active grain-filling period of grains at all locations on the ear and thereby decreased the grain weight. In contrast, PM had no significant effects on the active grain-filling period of the grains at the 270-mm and 320-mm irrigation levels. In addition, we found that the LAI and SPAD values for the PM treatment were significantly lower than those for the TN treatment at the mature stage at the 220-mm irrigation level. However, the LAI and SPAD values for the PM treatment were higher than those for the TN treatment at the mature stage at the 270-mm and 320-mm irrigation levels(data not shown). These results imply that at the 220-mm irrigation level, the maize plants in the PM treatment showed premature senescence. A previous study has suggested that PM may produce premature senescence because PM clearly increases the soil temperature [32, 33]. In the present study, we found that PM significantly increased the soil temperature at the 220-mm, 270-mm and 320-mm irrigation levels. However, premature senescence induced by PM was only apparent at the 220-mm irrigation level. We suggest that the reason for this difference is that PM significantly increased not only the soil temperature but also the soil moisture at the 220-mm, 270-mm and 320-mm irrigation levels; at the 270-mm and 320-mm irrigation levels, the higher soil moisture produced by the PM treatment moderated the effect of the high soil temperature on premature senescence. AT the 220-mm irrigation level, however, although PM significantly increased the soil moisture, the limited input of water was still sufficient to meet the need of the maize for water for growth. Accordingly, the increase in soil moisture did not moderate the effect of high soil temperature on premature senescence. As a result, the active grain-filling period for PM was markedly less than that for TN at the 220-mm irrigation level.

### Relationship between hormone changes and maze grain filling

Cytokinins (CTK) play an important role in regulating grain filling. It has been reported that CTK levels in rice spikelets are significantly correlated with seed development [34, 35]. In barley (*Hordeum vulgare* L.), maize, rice and wheat, high levels of cytokinins are generally found in the endosperm of developing seeds. These high levels may be required for cell division during the early phase of seed setting [18, 36–39]. In addition to CTK, IAA plays an important role in regulating grain filling [13, 14, 35]. The present study indicated that the IAA and Z+ZR content in the grains was positively and significantly correlated with the maximum grain weight and the maximum and mean grain-filling rates. This result implies that Z+ZR and IAA are involved in regulating grain filling in maize. In the present study, the changes in IAA and Z+ZR content in the grains showed a very similar pattern. The Z+ZR and IAA content in the grains transiently increased at the early grain-filling stage and then decreased, and the maximal IAA and Z+ZR content appeared just before the occurrence of the maximal grain-filling rate. Xu et al. [13] have suggested that CTK regulates endosperm cell division in developing rice grains. Davies [40] has stated that auxin also stimulates cell division. High IAA levels in the sink could create an ‘‘attractive power” leading to increased cytokinin levels in the grains [41, 42]. These results suggest that, similar to what is known for rice and wheat, IAA and Z+ZR may regulate maize grain filling at the early filling stage, most likely via manipulating the division of endosperm cells and thereby creating sink strength. Additionally, the correlation analysis performed in this study indicated that the IAA and Z+ZR content in the grains was positively and significantly correlated with the maximum grain weight and the maximum and mean grain-filling rates of the upper grains and middle grains. However, the IAA and Z+ZR content was not significantly correlated with the maximum grain weight and the maximum and mean grain-filling rates of the basal grains. This result implies that IAA and Z+ZR had a closer correlation with the grain filling of the upper and middle grains than with that of the basal grains. The reason for this difference may be that the grain-filling rate and grain weight were insensitive to PM and irrigation. The grain weight did not differ significantly between the PM and TN treatments at the 270-mm and 320-mm irrigation levels, and the mean grain weight for the PM and TN treatments also did not differ significantly between the 270-mm and 320-mm irrigation levels. However, PM and irrigation significantly affected the IAA and Z+ZR content in the basal grains. As a result, there was no significant correlation between the IAA and Z+ZR content and the grain-filling characteristics. These results also imply that although IAA and Z+ZR may regulate grain filling at the early filling stage in maize, these two hormones may not determine grain filling in maize.

In addition to Z+ZR and IAA, ABA and ETH play important roles in regulating grain filling. Yang et al. [19] have suggested that a higher ABA concentration and lower concentrations of ETH were associated with a higher filling rate in wheat grains. The regression analysis in the present study indicated that the ABA content in grains was positively and significantly correlated with the maximum grain weight and the maximum and mean grain-filling rates. In contrast, to this, the ETH evolution rate of the grains was negatively and significantly correlated with the maximum grain weight and the maximum and mean grain-filling rates. The same trend was also found for the upper grains, middle grains and basal grains. These results mean that ABA promotes grain filling in maize, whereas ETH inhibits grain filling in maize. This result was also obtained by a previous study [19].

Several reports have found that GAs are also involved in regulating grain development. Eeuwens and Schwabe [43] have stated that GA-like material was at its highest level in the liquid endosperm of pea at the time of rapid pod elongation. Relatively high levels of GA_1, 4, 19_ occur in the large panicle of rice just before and at anthesis [44, 45]. In the present study, the levels of GAs in the basal grains and middle grains were significantly less than those in the upper grains. However, the content of GAs in the grains was not significantly correlated with the maximum grain weight or the maximum and mean grain-filling rates. This result suggests that GAs may be involved in regulating grain filling in maize but may not be a determining factor for grain filling.

We found that IAA, Z+ZR and ABA are all involved in regulating grain filling. This result implies that the balance of hormones, rather than the content of individual hormones, regulates grain filling in maize. Moreover, PM regulated the balance of hormones and thereby affected grain filling. Further studies on the effects of individual hormones and of the interactions of hormones on the regulation of grain weight in maize are necessary.

## Conclusions

The effect of PM on maize grain filling is strongly related to the irrigation level and the position of the grains on the ear. At the 220-mm irrigation level, PM significantly increased the grain-filling rates of the middle and basal grains and significantly decreased the grain-filling rates of the upper grains. In addition, PM significantly decreased the active grain-filling period of grains at all positions on the ear at the 220-mm irrigation level. At the 270-mm irrigation level, the PM significantly increased the grain-filling rates of the upper grains, middle grains and basal grains. At the 320-mm irrigation level, PM significantly increased the grain-filling rate of the upper grains. PM and irrigation regulated hormonal changes, thereby regulating grain filling. ABA and ETH may play important roles in regulating the grain filling induced by PM in maize under different irrigation levels. In addition to IAA and Z+ZR are also involved in regulating the grain filling induced by PM in maize under different irrigation levels.
